# Potential Predictive Immune and Metabolic Biomarkers of Tumor Microenvironment Regarding Pathological and Clinical Response in Esophageal Cancer After Neoadjuvant Chemoradiotherapy: A Systematic Review

**DOI:** 10.1245/s10434-023-14352-z

**Published:** 2023-09-30

**Authors:** H. H. Wang, E. N. Steffens, G. Kats-Ugurlu, B. van Etten, J. G. M. Burgerhof, G. A. P. Hospers, J. T. M. Plukker

**Affiliations:** 1 Department of Medical Oncology, University of Groningen, University Medical Center Groningen, Groningen, The Netherlands; 2 Department of Pathology and Medical Biology, University of Groningen, University Medical Center Groningen, Groningen, The Netherlands; 3 Department of Surgical Oncology, University of Groningen, University Medical Center Groningen, Groningen, The Netherlands; 4 Department of Epidemiology, University of Groningen, University Medical Center Groningen, Groningen, The Netherlands

## Abstract

**Introduction:**

The tumor microenvironment (TME) plays a crucial role in therapy response and modulation of immunologic surveillance. Adjuvant immunotherapy has recently been introduced in post-surgery treatment of locally advanced esophageal cancer (EC) with residual pathological disease after neoadjuvant chemoradiotherapy (nCRT). F-18 fluorodeoxyglucose positron emission tomography/computed tomography (^18^F-FDG-PET/CT) remains a valuable imaging tool to assess therapy response and to visualize metabolic TME; however, there is still a paucity in understanding the interaction between the TME and nCRT response. This systematic review investigated the potential of TME biomarkers and ^18^F-FDG-PET/CT features to predict pathological and clinical response (CR) after nCRT in EC.

**Methods:**

A literature search of the Medline and Embase electronic databases identified 4190 studies. Studies regarding immune and metabolic TME biomarkers and ^18^F-FDG-PET/CT features were included for predicting pathological response (PR) and/or CR after nCRT. Separate analyses were performed for ^18^F-FDG-PET/CT markers and these TME biomarkers.

**Results:**

The final analysis included 21 studies—10 about immune and metabolic markers alone and 11 with additional ^18^F-FDG-PET/CT features. High CD8 infiltration before and after nCRT, and CD3 and CD4 infiltration after nCRT, generally correlated with better PR. A high expression of tumoral or stromal programmed death-ligand 1 (PD-L1) after nCRT was generally associated with poor PR. Moreover, total lesion glycolysis (TLG) and metabolic tumor volume (MTV) of the primary tumor were potentially predictive for clinical and PR.

**Conclusion:**

CD8, CD4, CD3, and PD-L1 are promising immune markers in predicting PR, whereas TLG and MTV are potential ^18^F-FDG-PET/CT features to predict clinical and PR after nCRT in EC.

**Supplementary Information:**

The online version contains supplementary material available at 10.1245/s10434-023-14352-z.

Standard curative treatment for potentially resectable locally advanced esophageal cancer (EC) (clinical TNM classification cT2-4a/Nany/M0) consists of neoadjuvant chemoradiotherapy (nCRT) followed by surgery according to the CROSS regimen.^1^ Although 20–30% of patients achieved a pathologic complete response (pCR), more than half still have residual tumor after nCRT.^2^ The tumor microenvironment (TME) is currently a focus in exploring additional treatment combinations. Several potential predictive markers have been evaluated to improve treatment outcome, including the expression of human epidermal growth factor receptor 2 (HER2) and programmed death-ligand 1 (PD-L1), as well as the prevalence of microsatellite instability (MSI). Based on the presence of MSI and PD-L1 expression among squamous cell (ESCC) and adenocarcinoma (EAC) of the esophagus, including the results of the landmark CheckMate-577 trial, adjuvant immunotherapy has been suggested in EC patients with residual pathologic disease.^3–10^ Differences in response of only immune checkpoint inhibitors have been observed in ESCC and EAC, which might be associated with important differences in the TME in upper/mid-esophageal (ESCC) and lower esophagus/gastroesophageal adenocarcinoma (EAC/GEA).^11–13^ Compared with EAC/GEA, ESCC exhibited a high expression of PD-L1 and a low HER-2 expression and high MSI (MSI-H) status.^14^

Therapy response and the activities of the TME are commonly visualized with F-18 fluorodeoxyglucose positron emission tomography/computed tomography (^18^F-FDG-PET/CT) scanning. ^18^F-FDG-uptake (glucose analog) measured by PET/CT indicates the highly increased glucose uptake because of the Warburg effect in tumor tissue. Many studies showed that the increased uptake of glucose and glycolysis by esophageal tumor cells might be caused by enhancement of membrane-bound glucose transporters (GLUT) and hexokinase (HK) enzymes.^15–17^ Studies have ever since tried to associate the Warburg effect in the tumor and its increased TME metabolic biomarkers with the semiquantitative standardized maximum uptake value (SUV_max_) in ^18^F-FDG-PET/CT.^18^

However, there is still a gap in our understanding of how the TME interacts with nCRT in EC. Therefore, we performed a systematic review to explore potential metabolic and immune TME biomarkers and their predictive role in pathological response (PR) and/or clinical response (CR) after nCRT in EC. As ^18^F-FDG-PET/CT may visualize the metabolic activity throughout the entire tumor, including its inflammatory microenvironment, it can be used to study the effect of additional immunotherapy in future studies. Combined with potent biomarkers, this metabolic imaging may be helpful in determining response to identify patients more likely to benefit from additional treatment or a potentially applicable organ-preserving treatment approach. Therefore, we also aimed to provide some future research perspectives on metabolic and immune TME biomarkers that might be associated with ^18^F-FDG-PET/CT (semi)-quantitative features.

## Materials and Methods

### Search Strategy and Study Selection Process

A systematic review according to the Preferred Reporting Items for Systematic Review and Meta-analysis Protocols (PRISMA-P) guidelines was performed.^19^ The study protocol was registered and the search strategy was documented online at the International Prospective Register of Systematic Reviews Registry (PROSPERO; ID CRD42022325532). The research question was to explore potential predictive immune and metabolic biomarkers in the interaction of nCRT and TME for a more effective treatment strategy. The exact search strategy is provided in electronic supplementary material (ESM) Table 1. The EMBASE and PubMed online databases were searched from 2001 until September 2022 using the following inclusion criteria: (1) original article/conference abstracts; (2) studies on ESCC or EAC and/or GEA; (3) published in peer-reviewed journals from 2001 or later; (4) studies on the effect of the metabolic, immune and PET-based TME on PR and/or CR after neoadjuvant treatment; and (5) studies published in English. The exclusion criteria were (1) studies with missing or unclear description/criteria for groups and/or variables; (2) if full text was not available; (3) studies not assessing CR after nCRT on pre- and post-treatment PET/CT; and (4) studies not including pathologic reports of the esophageal biopsy and PR of the surgical resection material.

**Table 1:** Main characteristics of selected studies

ID	Author	Journal	Year	Study design	*N*	TNM staging	Histology	CT/CRT/RT	Tissue type	Pathological score	Clinical score	Time of scan	Metabolic/immune markers
1	Göbel et al.^28^ ^a^	Clinical and Translational Radiation Oncology	2022	Retrospective	76	II–III	EAC, GEAC	CRT	Pretreatment biopsies	Mandard (TRG1–3 vs. TRG 4–5)	NA	NA	CD8, PD-1, PD-L1
2	Haddad et al.^29^ ^a^	Journal of Personalized Medicine	2022	Retrospective	43	I–III	EAC, GEAC, ESCC	CRT;CT	Surgical specimens	College of American Pathologist (1–3)	NA	NA	CD3+, CD4+, CD8+, CD45R0+, Foxp3+, CD20+, CD163+
3	Fassan et al. ^23^ ^a^	Cancer Medicine	2019	Retrospective	88	I–IV	ESCC	CRT	Surgical specimens	Mandard (TRG1 vs. TRG2–5)	NA	NA	CD4; Tbet; FoxP3; CD8; CD80; PD-L1;
4	Goedegebuure et al.^24^ ^a^	Oncoimmunology	2021	Retrospective	40	II–III	EAC, GEAC	CRT	Tumor samples; surgical specimens	Mandard (TRG1 vs. TRG 2–3 vs. TRG4–5)	NA	NA	CD8/CD163; CK: CD3; FoxP3; CD163; Ki67
5	Huang et al.^25^ ^a^	Journal of Clinical Medicine	2019	Retrospective	107	II–III	ESCC	CRT	Surgical specimens	pCR; no pCR	NA	NA	PD-L1
6	Koemans et al.^30^ ^a^	Histopathology	2021	Retrospective	123	II–III	EAC, GEAC	CRT	Surgical specimens	Mandard (TRG2 vs. TRG4–5)	NA	NA	CD3+, CD4+, CD8+, FOXP3, PD-L1
7	Kotsafti et al.^26^ ^a^	Oncoimmunology	2020	Prospective	123	II–III	EAC, GEAC	CRT	Pretreatment biopsies; healthy esophageal mucosa close to cancer site	Mandard (TRG1 vs. TRG2–5)	NA	NA	mRNA of CD80; CD8; CD28; CD38; CTLA4; CD8alpha; CD8beta; CD107a (LAMP1); CD69; Tbet (TBX21); SERPINB3; TP53; HER2 (ERBB2); PD-1 (PDCD1); PD-L1 (CD274); PD-L2 (PDCD1LG2); MLH1; MSH6; MSH3; PMS2; BRAF; IFNγ; FOXP3; CD25 (IL2RA); CD94 (KLRD1); CTLA4; TNFβ (LT; TNFSF1)
8	Soeratram et al.^27^ ^a^	The Journal of Pathology	2021	Retrospective	188	II–III	EAC, GEAC	CRT	Pretreatment biopsies	Mandard (TRG1–3 vs. 4–5)	NA	NA	PD-L1; PD-1; CD274PD-1; CD279; FOXP3; CD8; pan-cytokeratin multiplex; MHC class I, II duplex
9	Alvarado et al.^31^ ^a^	Seminars in Thoracic and Cardiovascular Surgery	2022	Retrospective	244	II–III	EAC, GEAC, ESCC	CRT	NA	pCR; no pCR	NA	NA	Diabetes
10	Boyd et al.^41^ ^a^	ASCO Annual Meeting I	2010	Retrospective	122	NA	EAC, GEAC, ESCC	CRT	NA	pCR; no pCR	NA	NA	DM; obesity
11	Fang et al.^32^ ^b^	International Journal of Radiation Oncology Biology Physics	2017	Prospective	20	IIa–IIIb	EAC; GEAC, ESCC	CRT	NA	Mandard (TRG1 vs. TRG2 vs. TRG3+)	ΔSUV_max_; ΔSUV_mean_; ΔTLG; MTV	Pre-CRT; during CRT; post-CRT	NA
12	Gillham et al.^33^ ^b^	British Journal of Cancer	2006	Retrospective	32	II–III	EAC, GEAC, ESCC	CRT	NA	Mandard (TRG1–2 vs. TRG3–5)	ΔSUV_mean_; MTV	Pre-CRT; during CRT	NA
13	Kukar et al.^34^ ^b^	JAMA Surgery	2015	Retrospective	77	NA	EAC, GEAC	CRT	NA	pCR; no pCR	SUV_max_; ΔSUV_mean_; %reduction SUV_max_; mean linear length of uptake, in cm, pre- and post-CRT; change in linear length	Pre-CRT; post-CRT	NA
14	Arnett et al.^35^ ^b^	International Journal of Radiation Oncology	2016	Retrospective	193	I–IV	EAC, GEAC	CRT	NA	pCR; no pCR	ΔSUV_max_; ΔSUR to blood pool uptake; ΔSUR to liver uptake	Pre-CRT; post-CRT	NA
15	Choi et al.^36^ ^b^	European Journal of Nuclear Medicine and Molecular Imaging	2021	Retrospective	480	0–III	ESCC	CRT	NA	Mandard (TRG1 vs. TRG2–5)	MTV; SUV_max_; SUV_avg_; TLG	Pre-CRT; post-CRT	NA
16	Dewan et al.^37^ ^b^	Journal of Gastrointestinal Cancer	2017	Prospective	70	I–III	ESCC	CRT	NA	pCR; no pCR	SUV_max_; % ΔSUV_max_	Pre-CRT; post-CRT	NA
17	Lee et al.^38^ ^b^	European Journal of Cardio-Thoracic Surgery	2021	Retrospective	158	I–III	ESCC	CRT	NA	pCR; no pCR	ΔSUV_max_; % ΔSUV_max_	Pre-CRT; post-CRT	NA
18	Piessen et al.^39^ ^b^	Annals of Surgery	2013	Retrospective	60	II–III	EAC, GEAC, ESCC	CRT	NA	Grade I: ≥50% VRTC; Grade II: 10–50% VRTC with fibrosis; Grade III: <10%^42^	% ΔSUV_max_	Pre-CRT; post-CRT	NA
19	Van Rossum et al.^40^ ^b^	European Journal of Nuclear Medicine and Molecular Imaging	2017	Retrospective	70		EAC, GEAC	CRT	NA	Chirieac’s TRG: TRG1–4 (TRG1–2 vs. TRG3–4)^43^	SUV_max_; SUV_mean_; MTV; TLG;	Pre-CRT; post-CRT	NA
20	Li et al.^21^ ^b^	Scientific Reports	2021	Retrospective	127	II–III	ESCC	CRT	Pre-CRT blood; post-CRT blood	pCR; no pCR	ΔSUV; ΔSUV ratio	Pre-CRT; post-CRT	NLR pre-CRT; NLR post-CRT
21	Wang et al.^22^ ^b^	European Journal of Cancer	2010	Retrospective	405	I–III	EAC, GEAC, ESCC, other	CRT	Surgical specimens	pCR; no pCR	NA	Pre-CRT; post-CRT	Obese (≥25 kg/m^2^); non-obese (< 25 kg/m^2^)

^a^ No presence of ^18^F-FDG-PET scan

^b^ Presence of ^18^F-FDG-PET scan

*CT* chemotherapy, *CRT* chemoradiotherapy, *RT* radiotherapy, *EAC* esophageal adenocarcinoma, *GEAC* gastroesophageal adenocarcinoma, *ESCC* esophageal squamous cell carcinoma, *pCR* pathologic complete response, *SUV*_max_ maximum standardized uptake value, *SUV*_mean_ mean standardized uptake value, *SUV*_avg_ average standardized uptake value, *TLG* total lesion glycolysis, *MTV* metabolic tumor volume, *SUR* standardized uptake ratio, *TRG* tumor regression grade, *NLR* neutrophil to lymphocyte ratio, *NA* not available, *PD-L1* programmed death-ligand 1, *PD-1* programmed death-1, *MHC* major histocompatibility complex, *DM* diabetes mellitus, ^*18F*^*-FDG-PET/CT* F-18 fluorodeoxyglucose positron emission tomography/computed tomography

### Quality Assessment

Risk of bias was assessed according to the study design and purpose. Non-randomized intervention studies were assessed using the Cochrane Risk of Bias in Nonrandomized Studies of Interventions (ROBINS-I) tool.^20^ All studies were evaluated with a visualization tool for risk-of-bias assessments in a systematic review (Risk-of-Bias VISualization Tool). Each article was read and assessed by two independent authors (HHW, ENS).

### Data Extraction and Synthesis

Two authors (HHW, ENS) extracted the data independently. Disagreements between individual judgments were resolved by discussion among the research group consisting of two surgical oncologists, one medical oncologist and one pathologist (all experienced) until consensus was reached. Data were recorded, extracted and managed in a Microsoft Excel spreadsheet (Microsoft Corporation, Redmond, WA, USA). The extraction and generation of the results were discussed together with a statistician (JGMB).

Relative and percentage ΔSUV, total lesion glycolysis (TLG) and metabolic tumor volume (MTV) changes were considered to be an index for CR on ^18^F-FDG-PET/CT scans.

## Results

### Identification of Studies

**Fig. 1:**
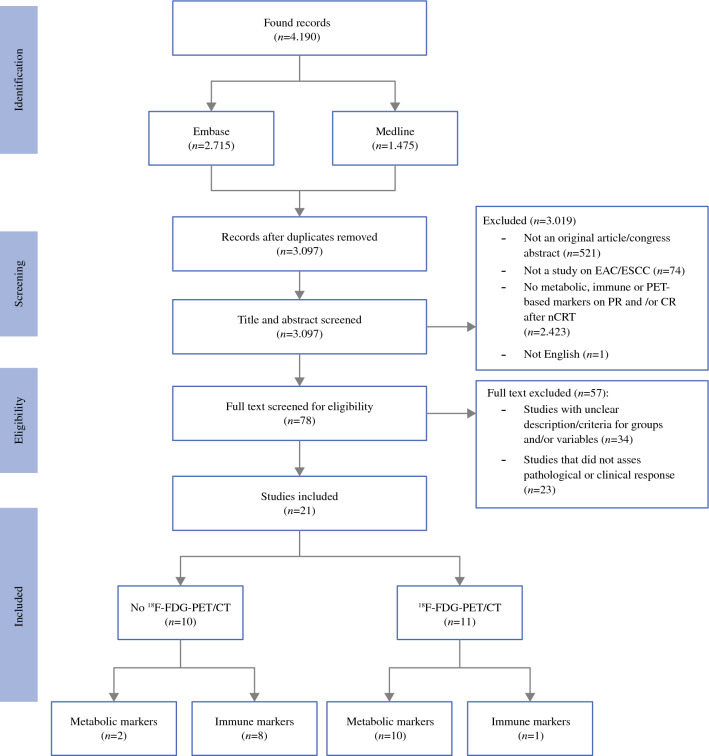
Screening of articles according to the PRISMA flowchart. *EAC* esophageal adenocarcinoma, *ESCC* esophageal squamous cell carcinoma, *PR* pathologic response, *CR* clinical response, *nCRT* neoadjuvant chemoradiotherapy, *PRISMA* Preferred Reporting Items for Systematic Reviews and Meta-Analyses, ^*18F*^*-FDG-PET/CT* F-18 fluorodeoxyglucose positron emission tomography/computed tomography

The initial electronic search identified 4190 studies. After eliminating duplicates, 3097 studies remained. These studies were screened using title and/or abstract to assess relevancy to our study scope. As both PR and CR were assessed, we distinguished between studies that included ^18^F-FDG-PET/CT scans and studies that did not. Seventy-eight articles were included for full screening (31 congress abstracts, 47 original articles); 57 were excluded due to unclear description/criteria for groups and/or variables (*n* = 34) or studies that did not assess PR and/or CR (*n* = 23). Finally, we included 21 studies (20 original articles^21–40^ and one study congress abstract^41^). We identified 10 studies on biological immune and metabolic TME biomarkers without the presence of an ^18^F-FDG-PET/CT scan (two studies on metabolic biomarkers, eight on immune biomarkers). Eleven studies were considered significant on clinical immune and metabolic TME biomarkers with the presence of an ^18^F-FDG-PET/CT scan (10 studies on metabolic biomarkers and 1 study on immune biomarkers) (Fig. 1).

### Study Characteristics

An overview of the study characteristics of the selected studies is provided in Table 1. Three studies^26,32,37^ were prospective and 18 studies were retrospective.^21–24,26–31,33–36, 38–41^ Eight studies assessed only EAC,^24,26–28,30, 34,35,40^ six studies assessed only ESCC,^21,23,25,36–38^ and seven studies included both types.^18–21,27,35,37^

All 21 studies assessed PR, of which 9 studies used the Mandard TRG scoring system,^23,24,26–28,30,32, 33,36^ 9 studies only assessed whether pCR was achieved^21,22,25,31,34,35, 37,38,41^ (defined as no viable tumor cells; ypT0), one study used the assessment of the College of American Pathologist (1–3),^29^ 1 study used pathologic grading according to Schneider et al.,^39,42^ and 1 study used pathologic grading according to Chirieac et al.^43^ Eight studies assessed immune markers, of which PD-L1, PD-1, CD80, CD8, CD4 and CD3 were assessed most extensively.^23–30^ Three studies assessed CD8, PD-L1 and PD-1 in diagnostic tumor biopsies before nCRT,^24,27,28^ while six studies assessed CD4, CD8, PD-L1, PD-1, CD80 and CD3 in surgical resection specimens after nCRT.^23–26,29,30^

Two studies determined whether diabetes mellitus (DM) affected pathologic outcome.^31,41^ In one of these studies, diabetic and non-diabetic patients were matched on patient and tumor characteristics.^31^ Both studies included both type 1 and type 2 DM.

All studies on CR included a baseline and post-nCRT ^18^F-FDG-PET scan. Eleven studies assessed CR,^21,22,32–40^ of which six studies assessed ΔSUV_max_,^21,32,34,35,38, 40^ four studies assessed percentage reduction SUV_max_,^34, 37–39^ four studies assessed mean tumor volume (MTV),^32, 33,36,40^ two studies assessed tumor lesion glycolysis (TLG),^36,40^ and three studies assessed ΔSUV_mean_.^32–34^

### Effect of Metabolic Markers on Pathologic Response

Two studies on the effect of DM on pathologic response were included and are shown in Table 2. In total, 73 diabetic patients and 293 non-diabetic patients were included. DM was associated with a decreased likelihood of achieving pCR according to Alvarado et al.,^31^ whereas Boyd et al. showed no significant difference between both groups.^41^

**Table 2:** Effect of metabolic marker diabetes on pathologic response (no ^18^F-FDG-PET/CT)

Author	Pathologic response	Diabetes (%)	No diabetes (%)	Total (%)	*p*-Value
Alvarado et al., 2022^31^ ^a^					**0.03**
	TRG1	5 (9.3)	41 (21.6)	46 (18.9)	
	TRG2–5	49 (90.7)	149 (78.4)	198 (81.1)	
Total		54 (100.0)	190 (100)	244 (100)	
Boyd et al., 2010^41^ ^a^					0.62
	TRG1	7 (36.8)	32 (31.1)	39 (10.7)	
	TRG2–5	12 (63.2)	71 (68.9)	83 (22.7)	
Total		19 (100.0)	103 (100.0)	122 (33.3)	

Bold value indicates the significant values (*p* < 0.05)

^a^ Univariate and multivariate regression

*TRG* tumor regression grade according to Mandard, ^*18F*^*-FDG-PET/CT* F-18 fluorodeoxyglucose positron emission tomography/computed tomography

### Effect of Immune Markers on Pathologic Response

Tables 3 and 4 show the pathologic immune markers on PR in treatment-naïve biopsies (Table 3) and surgical resections after nCRT (Table 4) in the primary tumor/TME/overall tumor area. Treatment-naïve biopsies were collected and assessed on immune markers prior to nCRT. The median density of immune markers was assessed in the total area.

**Table 3:** Effect of immune markers on pathologic response in the total area, tumor sample, and tumor microenvironment of treatment-naïve biopsies (no ^18^F-FDG-PET/CT)

Pre-CRT	Author	Assessment	Cut-off value	Sample size	*n* pGR (TRG1–3)	*n* pPR (TRG4–5)	Correlation biomarkers with PR in biopsies	*p*-Value total area	*p*-Value tumor	*p*-Value TME
*CD8*										
	Gobel et al., 2022^28^ ^a,b^	Cell count/mm^2^	Median	58	47	11	No significant difference in CD8 density between pGR and pPR	0.223	NA	NA
	Goedegebuure et al., 2021^24^ ^a,b^	Cell count/mm^2^	Median	40	31	9	High intratumoral density of CD8+ is associated with pGR No significant difference in stromal density between groups	NA	***0.031***	NM
	Soeratram et al., 2021^27^ ^c^	Cells/mm^2^	Mean and median	81	48	33	Higher combined mean density of CD8 was associated with pGR	**0.001**	**0.013**	**0.026**
*PD-1*										
	Gobel et al., 2022^28^ ^a,b^	<1%, 1 to <10%, 10 to <50%, ≥50%	–	58	47	11	Intra- and peritumoral PD-1 had no significant influence on TRG	NA	NM	NM
	Soeratram et al., 2021^28^ ^c^	cells/mm^2^	Mean and median	81	48	33	pPR had significant higher stromal PD-1 in lymphocytes compared with pGR	NA	0.222	**0.048**
*PD-L1*										
	Gobel et al., 2022^28^ ^a,b^	<1%, 1 to <10%, 10 to <50%, ≥50%	–	58	47	11	PD-L1-positive score in the tumoral area: no significant influence; PD-L1-positive score in the peritumoral area was found significantly more in pGR	NA	NM	**0.036**
	Soeratram et al., 2021^27^ ^c^	<1–4%, 5–24%, 25–100%	Mean and median	81	48	33	CPS > 1 was associated with lower TRG (1–3)	**0.010**	NA	NA

Bold values indicate the significant values (*p* < 0.05)

^a^ Pearson’s Chi-square test

^b^ Two-tailed *z*-test

^c^ Mann–Whitney *U*-test

^*18F*^*-FDG-PET/CT* F-18 fluorodeoxyglucose positron emission tomography/computed tomography, *TRG* tumor regression grade, *pGR* pathologic good responders, *pPR* pathologic poor responders, *PR* pathologic response, *CRT* chemoradiotherapy, *CPS* combined positive score, *PD-1* programmed death-1, *PD-L1* programmed death-ligand 1, *TME* tumor microenvironment, *NA* not available, *NM* not mentioned

**Table 4:** Effect of immune markers on pathologic response in the total area, tumor sample, and tumor microenvironment of surgical specimens (no ^18^F-FDG-PET/CT)

Post-CRT author	Assessment	Cut-off value	Sample size	*n* pGR (TRG1–2)^†^	*n* pPR (TRG3–5)^†^	Correlation biomarkers with PR in surgical specimens	*p*-Value total area	*p*-Value tumor	*p*-Value TME
*CD8*									
Fassan et al., 2019^44^ ^a,b^	Cell count/5 HPF	Median	88	23	65	pGR had significant enrichment of CD8+ compared with pPR	**<0.001**	NA	NA
Goedegebuure et al., 2021^24^ ^a^	Cell count/mm^2^	Median	40	12 (TRG1)	28 (TRG2–5)	TRG1 was significantly more present in a CD8-dominant infiltrate	**0.027**	NA	NA
Haddad et al., 2022^46^ ^c^	% ± SD	Mean	17	11	6	Significant enrichment of CD8+ in pGR compared with pPR	NA	**<0.001**	**0.001**
Koemans et al., 2021^30^ ^c^	Cell count/mm^2^	4 hotspots of 0.5 mm*0.5mm^2^	123	62	61	pGR had significantly less CD8+ compared with pPR	**0.001**	NA	NA
Kotsafti et al., 2021^26^ ^a,b^	Cell count/5 HPF	Median	123	20	103	Peritumoral healthy mucosa in pGR had significant high CD8+ compared with pPR	**0.05**	NA	NA
Soeratram et al., 2021^27^ ^a^	Cells/mm^2^	Mean and median	89	55 (TRG1–3)	25 (TRG4–5)	Higher CD8 in the tumor epithelium was associated with pPR; higher CD8 in tumor stroma was associated with pGR	NA	**0.000**	**0.000**
*CD4*									
Fassan et al., 2022^44^ ^a,b^	Cell count/5 HPF	Median	88	23	65	pGR had significant enrichment of CD4+ compared with pPR	**0.006**	NA	NA
Haddad et al., 2022^46^ ^c^	% ± SD	Mean	28	13	15	pGR had significant enrichment of CD4+ compared with pPR	NA	**0.009**	**0.004**
Koemans et al., 2021^30^ ^c^	Cell count/mm^2^	4 hotspots of 0.5 mm*0.5mm^2^	123	62	61	pGR had significantly less CD4+ compared with pPR	**<0.001**	NA	NA
*CD3*									
Haddad et al., 2022^46^ ^c^	% ± SD	Mean	88	13	15	pGR had significant enrichment of CD3+ compared with pPR	NA	**<0.001**	**<0.001**
Koemans et al., 2021^30^ ^c^	Cell count/mm^2^	4 hotspots of 0.5 mm*0.5mm^2^	123	62	61	pGR had significant less CD3+ compared with pPR	**<0.001**	NA	NA
*CD80*									
Fassan et al., 2022^44^ ^a,b^	Cell count/5 HPF	Median	88	23	65	No difference between pGR and pPR	0.4874	NA	NA
Kotsafti et al., 2021^26^ ^a,b^	Cell count/5 HPF	Median	123	20	103	No difference between pGR and pPR	0.89	NA	NA
*PD-1*									
Kotsafti et al., 2021^26^ ^a,b^	NA	–	123	20	103	pGR had significantly lower mRNA PD-1 compared with pPR	**0.0065**	NA	NA
*PD-L1*									
Fassan et al., 2019^44^ ^a,b^	Cell count/5 HPF	–	88	23	65	pGR had significant levels of PD-L1 expressions either on tumor cells or in lymphocytes than pPR	NA	**0.004**	NA
Huang et al., 2019^25^ ^d^	>1% = positive, <1 = negative	–	107	28	79	Positive tumoral PD-L1 expression was significantly associated with pPR	NA	**0.036**	NA
Koemans et al., 2021^30^ ^c^	0%, 1–5%, 6–9%, 10–29%, >30%	–	123	62	61	No association between PD-L1 positivity in tumor cells and PR; pPR had significantly more PD-L1-positive lymphocytes	NA	1.00	**0.001**
Kotsafti et al., 2021^26^ ^a,b^	NA	–	123	20	103	pGR had significantly lower mRNA PD-L1 compared with pPR	**0.0005**	NA	NA
Soeratram et al., 2021^27 a^	<1–4%, 5–24%, 25–100%	Mean and median	89	55 (TRG1–3)	25 (TRG4–5)	CPS > 1 was associated with pPR	**0.010**	NA	NA

Bold values indicate the significant values (*p* < 0.05)

^a^ Pearson’s Chi-square test

^b^ Two-tailed *z*-test

^c^ Mann–Whitney *U*-test

^d^ Logistic regression

^*18F*^*-FDG-PET/CT* F-18 fluorodeoxyglucose positron emission tomography/computed tomography, *TRG* tumor regression grade, *pGR* pathologic good responders, *pPR* pathologic poor responders, *PR* pathologic response, *CRT* chemoradiotherapy, *CPS* combined positive score, *TME* tumor microenvironment, *NA* not available, *PD-1* programmed death-1, *PD-L1* programmed death-ligand 1, *HPF* high power field, *SD* standard deviation

As the included studies combined different TRG groups, we were unable to create consistent TRG groups for this review. Tumor regression in these studies was based on vital tumor tissue at the ratio of fibrosis. In addition, patients with pCR (TRG1) were considered free of residual tumor, which is less likely compared with those with non-pCR (TRG2–5). Therefore treatment-naïve biopsies (Table 3) were divided according to the pathologic examination of the resected specimen in good (TRG1–3) and poor (TRG4–5) responders. The Mandard response rates from the treatment-naïve biopsies were extrapolated from their resected specimens. In assessing potential biomarkers in the resected specimen (Table 4), responders after nCRT were divided into pathologic good responders (TRG1–2) and pathologic poor responders (TRG3–5).

Table 3 shows that an overall higher tumoral and TME infiltration of CD8 in treatment-naïve biopsies was associated with a better PR (*p* = 0.013 and *p* = 0.026; *p* = 0.001; *p* = 0.031, respectively)^24,27^ Moreover, a higher PD-1 in the TME seemed to significantly predict the possible poor response in tumor tissue from treatment-naïve biopsies (*p* = 0.048) (Table 4); however, PD-1 in the primary tumor was shown to not be predictive for tumor response (*p* = 0.222) (Table 3).^27,28^ PD-L1 expression in the treatment-naïve biopsies showed to predict better PR (lower TRG) both in the TME as the overall tumoral and the TME area (*p* = 0.036, *p* = 0.010, respectively).^25,27^ Only Huang et al. showed that a high density of PD-L1 in the treatment-naïve biopsies predicted poor PR (higher TRG) (*p* = 0.036).^25^

Table 4 shows that tumoral and stromal CD8 was found to be significantly higher in pathologic good responders as well as in the healthy mucosa in resected specimens after nCRT.^26,44–46^ Soeratram et al., who distinguished tumoral and stromal CD8, showed that stromal CD8 was significantly associated with good pathologic response (*p* = 0.000, whereas tumoral CD8 was correlated with a poorer pathologic response (*p* = 0.000).^27^ Koemans et al. showed that good responders had significantly less CD8 in the overall area compared with poor responders after nCRT (*p* = 0.001).^30^

The majority of the studies found significant enrichment of CD4 in the tumor and the TME in surgical resection specimens after CRT (*p* = 0.006, *p* = 0.009, *p* = 0.004, respectively) (Table 4);^23,29^ however, one study contradicted these results and showed that poor responders had significant enrichment of CD4 density compared with poor responders (*p* ≤ 0.001).^30^

Furthermore, higher PD-1 in the overall tumor and stromal area was shown to be significantly predictive for a poor PR after nCRT (*p* = 0.0065).^26^

PD-L1 expression after nCRT proved to be associated with a poor PR according to Koemans et al. (*p* = 0.001).^30^ Moreover, a high PD-L1 in the overall area was correlated with a poor PR after nCRT (*p* = 0.0005, *p* = 0.010, respectively).^26,27^

Regarding CD80, two studies revealed no differences in CD80 between pathologic good and poor responders in the overall tumoral and stromal area after nCRT (*p* = 0.4874, *p* = 0.89, respectively).^26,44^

### Effect of Clinical Metabolic Markers on Pathological Response

We considered the semi-quantative tools that are used for measuring glucose metabolism and ^18^F-FDG uptake (SUV_max_, SUV_mean_, ΔSUV_max_ and percentage reduction SUV_max_) in the ^18^F-FDG-PET/CT scan as clinical metabolic markers. Table 5 describes the effect of ΔSUV_max_, percentage reduction SUV_max_, ΔSUV_mean_, TLG, MTV, and ΔSUV_ratio_ on pathologic response. Pathologic responders were divided into good responders (TRG1–2) and poor responders (TRG3–5).

**Table 5:** Effect of immune and metabolic markers on pathologic response (in the presence of ^18^F-FDG-PET/CT)

	Author	Total sample size	*n* pGR (TRG1–2)^a^	*n* pPR (TRG3–5)^a^	Correlation clinical biomarkers with pathologic tumor response	*p*-Value
Δ*SUV*_max_						
	Fang et al., 2017^32^ b	20	14	6	ΔSUV_max_ was not correlated with pCR on interim and post-CRT scan	0.508; 1.00
	Kukar et al., 2015^34^ ^c^	77	22	55	ΔSUV_max_ was higher in pGR	**0.03**
	Arnett et al., 2016^35^ ^d^	193	NM	NM	ΔSUV_max_ was not correlated with pCR	0.25
	Lee et al., 2021^38^ ^e^	158	44	114	ΔSUV_max_ did not differ between pCR and pPR	0.201
	Van Rossum et al., 2017^40^ ^d^	70	27	43	Higher ΔSUV_max_ was significantly related to a good response	**0.01**
	Li et al., 2021^21^ ^d^	127	57 (TRG1)	70 (TRG2–5)	ΔSUV_max_ was an independent predictor for pCR	**0.002**
*% reduction in SUV*_max_						
	Kukar et al., 2015^34^ ^c^	77	22	55	% reduction SUV_max_ was higher in pGR	**0.03**
	Lee et al., 2021^38^ ^e^	158	44	114	% reduction SUV_max_ did not differ between pCR and pPR	0.071
	Piessen et al., 2013^39^ ^b^	60	21	25	No significant difference in % reduction SUV_max_ between pGR and pPR	0.310
	Dewan et al., 2017^37^ ^d^	70	24 (TRG1)	46 (TRG2–5)	% reduction SUV_max_ of 72.32% predicts pCR (sensitivity 70.8%, specificity 67.4%)	**0.011**
*TLG*						
	Choi et al., 2021^36^ ^d^	275	75 (TRG1)	200 (TRG2–5)	Higher pre-CRT TLG (> 205.67) was associated with a lower probability of pCR	**0.0318**
	Van Rossum et al., 2017^40^ ^d^	70	27	43	Higher post-TLG was associated with a higher chance of pPR	**0.01**
*MTV*						
	Fang et al., 2017^32^ ^b^	20	14	6	MTV was not correlated with pCR on interim and post-CRT scan	0.198; 0.6
	Gillham et al., 2006^33^ ^c^	32	9	23	No correlation between MTV and TRG	0.472
	Choi et al., 2021^36^ ^d^	275	75 (TRG1)	200 (TRG2–5)	Higher post-MTV (> 4.99) was associated with a low probability of pCR	**0.0005**
	Van Rossum et al., 2017^40^ ^d^	70	27	43	Higher post-MTV was associated with a higher chance of pPR	**0.01**
*ΔSUVmean*						
	Fang et al., 2017^32^ ^b^	20	14	6	No correlation between ΔSUV_mean_ and TRG on interim and post-CRT scan	0.424; 0.704
	Gillham et al., 2006^33^ ^c^	32	9	23	No correlation between ΔSUV_mean_ and TRG	0.645
	Kukar et al., 2015^34^ ^c^	77	22	55	ΔSUV_mean_ was higher in pGR	**0.03**
*ΔSUVratio*						
	Li et al., 2021^21^ ^d^	127	57 (TRG1)	70 (TRG2–5)	ΔSUV_ratio_ was an independent predictor for pCR	**0.007**
*Obesity*						
	Wang et al., 2010^22^ ^d^	405	85 (TRG1)	121 (TRG2–5)	BMI is not a significant predictor for pCR	0.9879

Bold values indicate the significant values (*p* < 0.05)

^a^ In case studies that made different divisions in pathologic responders, the numbers and specific tumor regression grade were indicated in the table

^b^ Mann–Whitney test

^c^ Wilcoxon rank-sum test and Kruskal–Wallis

^d^ Logistic regression

^e^ Student’s *t*-test

^*18F*^*-FDG-PET/CT* F-18 fluorodeoxyglucose positron emission tomography/computed tomography, *pGR* pathologic good responders, *pPR* pathologic poor responders, *TRG* tumor regression grade according to Mandard, *SUV*_max_ maximum standardized uptake value, *SUV*_mean_ mean standardized uptake value, *TLG* total lesion glycolysis, *MTV* metabolic tumor volume, *SUV*ratio standardized uptake value ratio, *NM* not mentioned, *pCR* pathologic complete response, *BMI* body mass index

ΔSUV_max_ was evaluated in six studies.^21,32,34, 35,38,40^ Kukar et al. and van Rossum et al. showed that ΔSUV_max_ was higher in pathologic good responders (*p* = 0.03, *p* = 0.01, respectively).^34,40^ Moreover, Li et al. assessed ΔSUV_max_ as an independent predictor for pCR (*p* = 0.002).^21^ However, Arnett et al. and Lee et al. found no significant difference between ΔSUV_max_ in good and poor responders.^38,47^

Four studies assessed the effect of percentage reduction SUV_max_,^34,37–39^ of which two showed no significant difference between pathologic good and poor responders.^38,48^ Kukar et al. showed that pathologic good responders had a higher percentage reduction SUV_max_,^34^ while Dewan et al. set a cut-off of 72.32% reduction of SUV_max_ to be predictive for pCR.^37^

TLG was evaluated in two studies, showing that a high TLG before and after CRT was associated with poor PR (*p* = 0.0318, *p* = 0.01, respectively).^36,40^

Four studies assessed the effect of MTV,^32,33,36,40^ of which two showed that a high post-CRT MTV was correlated with a poor PR (*p* = 0.0005, *p* = 0.01, respectively).^36,40^ The other two studies showed no correlation with PR (*p* = 0.6, *p* = 0.472, respectively).^32,33^

ΔSUV_mean_ was assessed in three studies, of which two showed no correlation between pathologic response.^32–34^ However, Kukar et al. assessed that pathologic good responders had a higher ΔSUV_mean_ compared with poor responders (*p* = 0.03).^34^

Only one study evaluated body mass index on PR, which showed no significant prediction for pCR (*p* = 0.9879).^22^

### Effect of Metabolic and Immune Markers on Clinical Response and Pathologic Response (^18^F-FDG-PET/CT)

ESM Table 2 shows the effect of immune and metabolic markers on PR and CR. Both studies divided the assessed groups into pCR (TRG1) or no pCR (TRG2–5).

Wang et al. evaluated the effect of obesity as a metabolic marker on CR, which showed not to be a significant predictor (*p* = 0.46).^22^ Li et al. assessed the correlation between immune markers neutrophil to lymphocyte ratio (NLR) and PET markers on prediction of PR, which showed that ΔNLR <3 and ΔSUV_ratio_ >58% gave the best positive predictive value (84.8%) for pCR.^21^

### Risk-of-Bias Assessment

Risks of bias was assessed for all included studies (*n* = 21) [ESM Fig. 1]. The individual risk-of-bias scores can be found in ESM Table 3 and ESM Table 4, on each risk of bias for each included study separately.

## Discussion

Metabolic and immune biomarkers of the TME have a pivotal role in providing tumor cells the optimal condition to survive and proliferate while also influencing their response to therapy. Due to intratumoral and microenvironmental heterogeneity after nCRT, all available information from the tumor, its TME, and the pathological specimen was included. Here, we provide an overview of potential metabolic and immune TME biomarkers that might play a role in PR and CR after nCRT in EC.

Current research in targeting the metabolic TME is based on ^18^F-FDG-PET/CT imaging of the altered glycolytic tumor metabolism with acidification of the TME. TME acidification induces hypoxia response pathways and leads to evasion of the immune system, which is associated with high metastatic potential and treatment resistance.^49^ As such, the upregulation of glycolysis as a measure of extracellular acidification remains a critical step in the activation of immune cells. In this intricate interaction of heterogeneous tumor cells, a variety of secretory cytokines and chemokines from non-malignant cells, i.e., stroma and immune cells, are involved in the efficacy of anticancer therapy. Metabolic remodeling with inflammatory response and oxidative phosphorylation is important in the resistance to neoadjuvant treatment in EC. Recently, a promising novel ex vivo method showed the significance of oxidative phosphorylation in measuring real-time metabolic profiles of treatment-naïve EC biopsies. In clinical imaging of hypoxic response and glycolytic metabolism in malignant tumors, ^18^F-FDG-PET/CT is most commonly used.^50^ Based on the assessment of histopathology, the corresponding ^18^F-FDG-PET/CT response and promising biomarkers markers, nCRT combined with immunotherapy might be considered as an organ-preserving treatment approach in the near future.

### Metabolic Tumor Microenvironment (TME) Markers

There were no studies on metabolic TME markers in EC that also assessed the influence of these markers on PR and/or CR after nCRT. Diabetes was suggested as a surrogate metabolic marker. However, the result of this study shows a limited role of DM on PR after nCRT. An overexpression of insulin receptors and insulin-like growth factors lead to the promotion of cell cycle progression and inhibition of apoptosis.^51,52^ The overexpressed insulin receptors on cancer cells of diabetic patients, who are also characterized by hyperinsulinemia, may be activated, leading to the ability of cancer cells to evade destruction by chemoradiotherapy, resulting in an unfavorable PR and CR.^53^ As a result, hypoxia and hyperglycemia occur, which might help remodeling the TME into an even more aggressive environment, leading to poorer response to nCRT.^54^

### Immune TME Biomarkers

We showed that high CD3 and CD4 infiltration were generally correlated with better PR. Even though some studies showed no significant difference in CD8 between good and poor pathologic responders, CD8 infiltration in treatment-naïve biopsies was generally significantly associated with a better PR.^24,27^ One study showed that nCRT was useful to induce CD4 and CD8 infiltration within the TME, suggesting that an elevated level of lymphocytes before nCRT might be a surrogate of a strong immune response induced by tumor cell necrosis caused by chemotherapy.^55^

The activation of CD8 cells after nCRT might be impaired by persistent high expression of the CXCL12/CXCR4 axis in EC stem cells resulting in a downregulation of major histocompatibility complex (MHC) class I molecules and upregulating immunosuppressive cytokines.^56^ nCRT can also cause inflammation, leading to an influx of CD8 immune cells. These patients could benefit from the upcoming immune-directed treatment strategies such as PD-1/PD-L1 blockade.^57–60^ In this study, CD8 pre/post-nCRT and CD3/CD4 after nCRT seem to be involved in the antitumor response. Moreover, the location (i.e., tumoral or stromal) at which the CD8 influx occurs might affect active immune behavior. The extracellular matrix or other immune-suppressive cells within the tumor and the TME might barricade the function of tumoral CD8,^61,62^ resulting in an inefficient function of CD8 intratumorally.

The potential clinical value of tumoral PD-L1 expression in EC patients with residual disease after nCRT with surgery has shown to be significant for DFS after adjuvant anti-PD-1 nivolumab in the Checkmate-577 study, and showed a better PR in the Keynote-590 study with anti-PD-1 pembrolizumab and nCRT.^3,7^ The included studies also showed that a high proportion of PD-L1 in positive treatment-naïve tumor samples may affect PR; however, the exact mechanism behind this is still unknown. PD-L1 expression in pretreatment biopsies might be different due to intratumoral heterogeneity of EC, in which PD-L1 expression can only be partially captured. However, further investigation is needed.

We also showed that a higher expression of tumoral or stromal PD-L1 after nCRT is generally associated with a poor PR to chemoradiotherapy. Therefore, PD-L1 might be a potential target in EC patients receiving nCRT in order to improve therapy response. Together with the other predictive immune biomarkers, PD-L1 expression in the tumor and its microenvironment could be used to define EC patients with major or poor pathologic response after nCRT with resection and/or a clinical prognostic high- versus low-risk profile. PD-L1 positivity can be expressed by using both the tumor cell (TC ≥ 1% in at least 100 tumor cells in the PD-L1-stained slide) and combined positivity score (CPS ≥10 PD-L1-stained cells, including tumor cells, lymphocytes, macrophages in the associated infiltration). Based on the histologic EC subtypes, these clinical prognostic risk biomarkers and the different predictive response biomarkers between tumor-naïve biopsies and the resected residual tumor material potential biomarkers may be identified for the ypCR and non-ypCR groups.

### ^18^F-FDG-PET/CT Biomarkers

An ^18^F-FDG-PET/CT scan is commonly used in EC patients undergoing the CROSS regimen, to monitor treatment response. Many studies aimed to find a correlation between the semi-quantitative parameters of ^18^F-FDG-PET/CT with PR. However, our included studies showed contradictory evidence for the value of parameters such as SUV, ΔSUV_max_ and SUV_max_ in predicting PR and CR.

A low SUV might be associated with hypoxic tumors, as is the case in EC. An hypoxic environment could emerge if the tumor became more resistant to chemoradiotherapy, leading to a poor pathologic response.^63^ Moreover, a wide heterogeneity between studies could account for contradictory results, such as different methods and experience at performing and interpreting ^18^F-FDG-PET/CT scans, methods to calculate PET parameters, physiological factors that may affect SUV uptake (i.e., inflammation) to the esophageal mucosa, scanner technology, chemoradiotherapy schedules, sample size, and methods of data collection. Studies also vary regarding the time interval of post-treatment ^18^F-FDG-PET/CT after completion of nCRT, which may affect the interpretation of predictive accuracy.

Therefore, the predictive value of other clinical ^18^F-FDG-PET/CT-based markers needs to be explored. We showed that TLG and MTV might have more potential to predict pathological and clinical outcome, which is also in line with recent studies.^64,65^ These volume-based ^18^F-FDG-PET parameters might provide more valuable information that supplement SUV uptake for predicting PR and CR. Future studies should thus focus on combining these parameters and find a clear cut-off value.

The present study has some limitations. Treatment-naïve EC biopsies contain a highly heterogenous inflammatory secretion profile. It is plausible that pretreatment-naïve biopsies are not representative enough. Therefore, it is important to know which specimen has been used in determining the predictive role of biomarkers. First, tumor heterogeneity may be missed in these small standard diagnostic tumor biopsies, and second, we should be aware of changes in biomarkers during chemotherapy and/or radiotherapy.^66^ Furthermore, biology from resected tissue alone may not reflect tumor biology at diagnosis. Moreover, patients attaining pCR (ypT0/N0) who commonly exhibit a good prognosis will not likely receive adjuvant therapy. Furthermore, we included articles of various markers that were assessed in different ways, i.e., mRNA expression of assessed markers, assessments conducted in healthy esophageal mucosa, and overall density of assessed markers. These differences in assessing various markers made it difficult to interpret the results.

## Conclusion

Our systematic review showed that CD8, CD4, CD3, and PD-L1 are promising immune markers in predicting PR. Moreover, we showed that TLG and MTV have potential in predicting CR and PR. Additional research should focus more on combining histopathology and nuclear imaging features in EC before and after nCRT to assess metabolic and immune TME markers.

### Supplementary Information

Below is the link to the electronic supplementary material.

Supplementary file1 (DOCX 1722 KB)
