# Supplementary material for: Potential Predictive Immune and Metabolic Biomarkers of Tumor Microenvironment Regarding Pathological and Clinical Response in Esophageal Cancer After Neoadjuvant Chemoradiotherapy: A Systematic Review
Source: Ann Surg Oncol. 2023 Sep 30;31(1):433–51. doi: 10.1245/s10434-023-14352-z (PMC10695872; doi:10.1245/s10434-023-14352-z)
Supplement: Supplementary file 1 — Supplementary file1 (DOCX 1722 KB) [file 10434_2023_14352_MOESM1_ESM.docx]

**Supplementary table 1. Search strategies**

| **Pubmed** | |
| --- | --- |
| **Search strategy** | **Search terms** |
| 1. **Esophageal carcinoma** | (“esophageal neoplasms”[MeSH Terms] OR “esophageal carcin*”[tiab] OR “esophageal neoplasm*”[tiab] OR Esophageal cancer*[tiab] OR esophageal tum*[tiab] OR esophageal squamous cell carcinoma*[tiab] OR esophageal adenocarcinoma*[tiab] OR cancer of the esophagus[tiab] OR tumor of the esophagus [tiab] OR tumour of the esophagus [tiab] OR “oesophageal carcin*”[tiab] OR “oesophageal neoplasm*”[tiab] OR oesophageal cancer*[tiab] OR oesophageal tum*[tiab] OR oesophageal squamous cell carcinoma*[tiab] OR oesophageal adenocarcinoma [tiab] OR cancer of the oesophagus[tiab] OR tumor of the oesophagus[tiab] OR tumour of the oesophagus[tiab]) |
| 1. **Neoadjuvant chemoradiotherapy** | ("neoadjuvant therapy"[MeSH Terms] OR "chemoradiotherapy"[MeSH Terms] OR "neoadjuvant therap*"[Title/Abstract] OR neoadjuvant treatment* [tiab] OR neo-adjuvant therap*[tiab] OR neo-adjuvant treatment* [tiab] OR “chemoradiotherap*”[tiab] OR chemo-radiotherap*[tiab] OR radiotherap*[tiab] OR chemotherap*[tiab] OR chemo-therap*[tiab] OR neoadjuvant radiation*[tiab] OR neo-adjuvant radiation*[tiab]OR chemoradiation*[tiab] OR chemo-radiation*[tiab] OR Radiochemotherap*[tiab] OR radio-chemotherap*[tiab]) |
| 1. **Tumor microenvironment** | ("tumor microenvironment"[MeSH Terms] OR microenvironment*[Title/Abstract] OR epithelial mesenchymal transformation*[Title/Abstract] OR "epithelial mesenchymal transition"[MeSH Terms] OR epithelial mesenchymal transition*[tiab] OR EMT [tiab] OR extracellular matrix*[tiab] OR ECM[tiab] OR aerobic glycolysis [tiab] OR stromal*[tiab] OR environment*[tiab] OR metabol*[tiab] OR immun*[tiab] OR Warburg [tiab] OR "Biomarkers"[Mesh] OR biomarker*[tiab] OR marker*[tiab] OR "lymphocytes, tumor infiltrating"[MeSH Terms] OR "tumor infiltrating lymph*” [Title/Abstract] OR tumour infiltrating lymph*[tiab] OR "Cancer-Associated Fibroblasts"[Mesh] OR cancer-associated fibroblast*[tiab] OR tumor associated fibroblast*[tiab] OR tumour associated fibroblast*[tiab] OR antigen* [tiab] OR "Fluorodeoxyglucose F18"[Mesh] OR "Positron-Emission Tomography"[Mesh] OR PET-CT [tiab] OR petct[tiab] OR Positron Emission Tomography-Computed Tomograph* [tiab] OR CT PET [tiab] OR 18F-FDG [tiab] OR 18F-Fluorodeoxyglucose [tiab] OR Fluorine-18-fluorodeoxyglucose [tiab] OR 18FDG [tiab] OR PET-scan*[tiab] OR Positron Emission Tomograph*[tiab] OR PET imag*[tiab] OR “lipids”[Mesh] OR lipoprotein*[tiab] OR HDL [tiab] OR LDL [tiab] OR “body mass index”[Mesh] OR bmi[tiab]) |
| 1. **Pathologic and clinical response** | ("Neoplasm Grading"[Mesh] OR tumor grading [tiab] OR tumour grading [tiab] OR pathologic respon* [tiab] OR pathological respon*[tiab] OR pathologic complete respon*[tiab] OR pathological complete respon* [tiab] OR tumor regression grad* [tiab] OR tumour regression grad* [tiab] OR Mandard [tiab] OR Becker [tiab] OR Chirieac [tiab] OR Cologne [tiab] OR Schneider [tiab] OR treatment outcome [Mesh] OR treatment outcome* [tiab] OR partial respon*[tiab] OR pcr [tiab] OR pT0 [tiab] OR tumor regression scor* [tiab] OR tumor shrink*[tiab] OR tumour shrink* [tiab] OR tumor fragmentation* [tiab] OR tumour fragmentation* [tiab] OR clinical respon* [tiab]) |
| **Embase** | |
| 1. **Esophageal carcinoma** | ('esophagus cancer'/exp OR ‘esophageal carcin*’:ab,ti,kw OR ‘esophageal neoplasm*’:ab,ti,kw OR ‘esophageal cancer*’:ab,ti,kw OR ‘esophageal tum*’:ab,ti,kw OR ‘esophageal squamous cell carcinoma*’:ab,ti,kw OR ‘esophageal adenocarcinoma*’:ab,ti,kw OR ‘cancer of the esophagus’:ab,ti,kw OR ‘tumor of the esophagus’:ab,ti,kw OR ‘tumour of the esophagus’:ab,ti,kw OR ‘oesophageal carcin*’:ab,ti,kw OR ‘oesophageal neoplasm*’:ab,ti,kw OR ‘oesophageal cancer*’:ab,ti,kw OR ‘oesophageal tum*’:ab,ti,kw OR ‘oesophageal squamous cell carcinoma*’:ab,ti,kw OR ‘oesophageal adenocarcinoma’:ab,ti,kw OR ‘cancer of the oesophagus’:ab,ti,kw OR ‘tumor of the oesophagus’:ab,ti,kw OR ‘tumour of the oesophagus’:ab,ti,kw) |
| 1. **Neoadjuvant chemoradiotherapy** | ('neoadjuvant therapy'/exp OR 'chemoradiotherapy'/exp OR ‘neoadjuvant therap*’:ab,ti,kw OR ‘neoadjuvant treatment*’:ab,ti,kw OR ‘neo-adjuvant therap*’:ab,ti,kw OR ‘neo-adjuvant treatment*’:ab,ti,kw OR ‘chemoradiotherap*’:ab,ti,kw OR ‘chemo-radiotherap*’:ab,ti,kw OR ‘radiotherap*’:ab,ti,kw OR ‘chemotherap*’:ab,ti,kw OR ‘chemo-therap*’:ab,ti,kw OR ‘neoadjuvant radiation*’:ab,ti,kw OR ‘neo-adjuvant radiation*’:ab,ti,kw OR ‘chemoradiation*’:ab,ti,kw OR ‘chemo-radiation*’:ab,ti,kw OR ‘Radiochemotherap*’:ab,ti,kw OR ‘radio-chemotherap*’:ab,ti,kw) |
| 1. **Tumor microenvironment** | ('tumor microenvironment'/exp OR ‘microenvironment*’:ab,ti,kw OR ‘epithelial mesenchymal transformation*’:ab,ti,kw OR 'epithelial mesenchymal transition'/exp OR ‘epithelial mesenchymal transition*’:ab,ti,kw OR ‘EMT’:ab,ti,kw OR ‘extracellular matrix*’:ab,ti,kw OR ‘ECM’:ab,ti,kw OR ‘aerobic glycolysis’:ab,ti,kw OR ‘stromal*’:ab,ti,kw OR ‘environment*’:ab,ti,kw OR ‘metabol*’:ab,ti,kw OR ‘immun*’:ab,ti,kw OR ‘Warburg’:ab,ti,kw OR ‘Biomarkers’:ab,ti,kw OR ‘biomarker*’:ab,ti,kw OR ‘marker*’:ab,ti,kw OR 'tumor associated leukocyte'/exp OR ‘tumor infiltrating lymph*’:ab,ti,kw OR ‘tumour infiltrating lymph*’:ab,ti,kw OR 'cancer associated fibroblast'/exp OR ‘cancer-associated fibroblast*’:ab,ti,kw OR ‘tumor associated fibroblast*’:ab,ti,kw OR ‘tumour associated fibroblast*’:ab,ti,kw OR ‘antigen*’:ab,ti,kw OR 'fluorodeoxyglucose f 18'/exp OR 'positron emission tomography'/exp OR ‘PET-CT’:ab,ti,kw OR ‘petct’:ab,ti,kw OR ‘Positron Emission Tomography-Computed Tomograph*’:ab,ti,kw OR ‘CT PET’:ab,ti,kw OR ‘18F-FDG’:ab,ti,kw OR ‘18F-Fluorodeoxyglucose’:ab,ti,kw OR ‘Fluorine-18-fluorodeoxyglucose’:ab,ti,kw OR ‘18FDG’:ab,ti,kw OR ‘PET-scan*’:ab,ti,kw OR ‘Positron Emission Tomograph*’:ab,ti,kw OR ‘PET imag*’:ab,ti,kw OR ‘lipids’/exp OR ‘lipoprotein*’:ab,ti,kw OR ‘HDL’:ab,ti,kw OR ‘LDL’:ab,ti,kw OR ‘body mass index’/exp OR ‘bmi’:ab,ti,kw) |
| 1. **Pathologic and clinical response** | ('cancer grading'/exp OR ‘tumor grading’:ab,ti,kw OR ‘tumour grading’:ab,ti,kw OR ‘pathologic respon*’:ab,ti,kw OR ‘pathological respon*’:ab,ti,kw OR ‘pathologic complete respon*’:ab,ti,kw OR ‘pathological complete respon*’:ab,ti,kw OR ‘tumor regression grad*’:ab,ti,kw OR ‘tumour regression grad*’:ab,ti,kw OR ‘Mandard’:ab,ti,kw OR ‘Becker’:ab,ti,kw OR ‘Chirieac’:ab,ti,kw OR ‘Cologne’:ab,ti,kw OR ‘Schneider’:ab,ti,kw OR 'treatment outcome'/exp OR ‘treatment outcome*’:ab,ti,kw OR ‘partial respon*’:ab,ti,kw OR ‘pcr’:ab,ti,kw OR ‘pT0’:ab,ti,kw OR ‘tumor regression scor*’:ab,ti,kw OR ‘tumor shrink*’:ab,ti,kw OR ‘tumour shrink*’:ab,ti,kw OR ‘tumor fragmentation*’:ab,ti,kw OR ‘tumour fragmentation*’:ab,ti,kw OR ‘clinical respon*’:ab,ti,kw) |

**Supplementary table 2. The effect of metabolic and immune markers on clinical response pathologic response.** TRG = tumor regression grade; pCR = pathologic complete response; cGR = clinical good responder; cPR = clinical poor responder; CR = clinical response; PPV = positive predictive value; NLR = neutrophil lymphocyte ratio.

|  | **Author** | **Total sample size** | ***n* pCR (TRG1)** | ***n* no pCR (TRG2-5)** | **Correlation clinical biomarkers on clinical response and/or pathologic response** | **P-value cGR-cPR** |
| --- | --- | --- | --- | --- | --- | --- |
| **Obesity** | Wang et al. 2010^30^* | 405 | 85 | 121 | BMI is not a significant predictor for CR;  BMI is not a significant predictor for pCR | 0.46  0.9879 |
| **NLR** | Li et al. 2021^29^* | 127 | 57 | 70 | ΔNLR<3 and ΔSUV ratio>58% gave best PPV (84.8%) for pCR | NA |

***Multivariate logistic regression model**


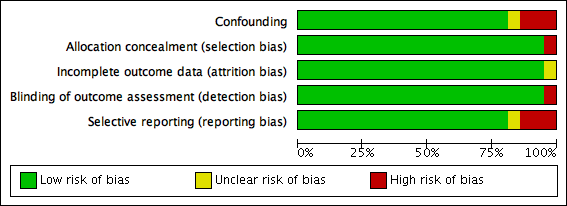


**Supplementary figure 1.** The risk of bias graph: review authors’ judgement about each risk of bias item presented as percentages across all included studies.

**Supplementary table 3. Risk of bias summary: review authors' judgements about each risk of bias item for each included study.**


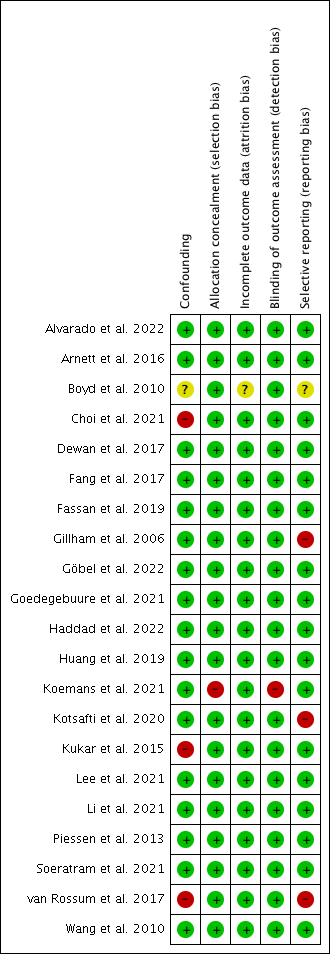


**Supplementary table 4. Risk of bias for each individual included study**

| Alvarado et al. 2022 | Risk of bias | Author judgement |
| --- | --- | --- |
| Confounding | Low risk | No confounding expected because they controlled for confounding factors |
| Allocation concealment (selection bias) | Low risk | All participants who were eligible in the study were included |
| Incomplete outcome data (attrition bias) | Low risk | Data was complete |
| Blinding of outcome assessment (detection bias) | Low risk | Methods of outcome assessment were comparable between both groups; the outcome measure was unlikely to be influenced by knowledge of the intervention received by study participants; |
| Selective reporting (reporting bias) | Low risk | All reported results correspond to all intended outcomes |
| Arnett et al. 2016 | Risk of bias | Author judgement |
| Confounding | Low risk | No confounding expected |
| Allocation concealment (selection bias) | Low risk | All participants who would have been eligible for the target trial were included in the study; For each participant, start of follow up and start of intervention coincided |
| Incomplete outcome data (attrition bias) | Low risk | Data was complete |
| Blinding of outcome assessment (detection bias) | Low risk | The methods of outcome assessment were comparable across intervention groups; The outcome measure is only minimally influenced by knowledge of the intervention received by study participants |
| Selective reporting (reporting bias) | Low risk | All reported results correspond to all intended outcomes |
| Boyd et al. 2010 | Risk of bias | Author judgement |
| Confounding | Unclear risk | No information on whether confounding might be present |
| Allocation concealment (selection bias) | Low risk | All participants who were eligible in the study were included |
| Incomplete outcome data (attrition bias) | Unclear risk | No information is reported about missing data or the potential for data to be missing. |
| Blinding of outcome assessment (detection bias) | Low risk | Methods of outcome assessment were comparable between both groups; the outcome measure was unlikely to be influenced by knowledge of the intervention received by study participants; |
| Selective reporting (reporting bias) | Unclear risk | There is insufficient information to make a judgement (as it is a congress abstract) |
| Choi et al. 2010 | Risk of bias | Author judgement |
| Confounding | High risk | The intervals between the FDG PETCT before and after nCRT differed between patients, which were not controlled for in the analyses |
| Allocation concealment (selection bias) | Low risk | All participants who would have been eligible for the target trial were included in the study. For each participant, start of follow up and start of intervention coincided |
| Incomplete outcome data (attrition bias) | Low risk | Data was complete |
| Blinding of outcome assessment (detection bias) | Low risk | Methods of outcome assessment were comparable between both groups; the outcome measure was unlikely to be influenced by knowledge of the intervention received by study participants (blinded) |
| Selective reporting (reporting bias) | Low risk | All reported results correspond to all intended outcomes |
| Dewan et al. 2017 | Risk of bias | Author judgement |
| Confounding | Low risk | No confounding expected |
| Allocation concealment (selection bias) | Low risk | All participants who were eligible in the study were included |
| Incomplete outcome data (attrition bias) | Low risk | Data was complete |
| Blinding of outcome assessment (detection bias) | Low risk | Methods of outcome assessment were comparable between both groups; the outcome measure was unlikely to be influenced by knowledge of the intervention received by study participants |
| Selective reporting (reporting bias) | Low risk | There is no indication of selection of the reported analysis from among multiple analyses; The outcome measurements are clearly defined and both internally and externally consistent |
| Fang et al. 2017 | Risk of bias | Author judgement |
| Confounding | Low risk | No confounding expected |
| Allocation concealment (selection bias) | Low risk | All participants who would have been eligible for the target trial were included in the study; For each participant, start of follow up and start of intervention coincided |
| Incomplete outcome data (attrition bias) | Low risk | Data was complete |
| Blinding of outcome assessment (detection bias) | Low risk | The methods of outcome assessment were comparable across intervention groups; The outcome measure is only minimally influenced by knowledge of the intervention received by study participants |
| Selective reporting (reporting bias) | Low risk | All reported results correspond to all intended outcomes |
| Fassan et al. 2019 | Risk of bias | Author judgement |
| Confounding | Low risk | No confounding expected |
| Allocation concealment (selection bias) | Low risk | All participants who would have been eligible for the target trial were included in the study. For each participant, start of follow up and start of intervention coincided |
| Incomplete outcome data (attrition bias) | Low risk | Data was complete |
| Blinding of outcome assessment (detection bias) | Low risk | The methods of outcome assessment were comparable across intervention groups; The outcome measure is only minimally influenced by knowledge of the intervention received by study participants |
| Selective reporting (reporting bias) | Low risk | All reported results correspond to all intended outcomes |
| Gillham et al. 2006 | Risk of bias | Author judgement |
| Confounding | Low risk | No confounding expected |
| Allocation concealment (selection bias) | Low risk | All participants who would have been eligible for the target trial were included in the study. For each participant, start of follow up and start of intervention coincided |
| Incomplete outcome data (attrition bias) | Low risk | Data was complete |
| Blinding of outcome assessment (detection bias) | Low risk | The outcome assessor (1 blinded pathologist) was unaware of the intervention received by study participants |
| Selective reporting (reporting bias) | High risk | Outcomes are grouped in the results, which was not mentioned in the methods |
| Göbel et al. 2022 | Risk of bias | Author judgement |
| Confounding | Low risk | No confounding expected |
| Allocation concealment (selection bias) | Low risk | All participants who would have been eligible for the target trial were included in the study |
| Incomplete outcome data (attrition bias) | Low risk | Data was complete |
| Blinding of outcome assessment (detection bias) | Low risk | The methods of outcome assessment were comparable across intervention groups; The outcome measure is only minimally influenced by knowledge of the intervention received by study participants |
| Selective reporting (reporting bias) | Low risk | All reported results correspond to all intended outcomes |
| Goedegebuure et al. 2021 | Risk of bias | Author judgement |
| Confounding | Low risk | No confounding expected |
| Allocation concealment (selection bias) | Low risk | All participants who would have been eligible for the target trial were included in the study. For each participant, start of follow up and start of intervention coincided |
| Incomplete outcome data (attrition bias) | Low risk | Data was complete |
| Blinding of outcome assessment (detection bias) | Low risk | The methods of outcome assessment were comparable across intervention groups; The outcome measure is only minimally influenced by knowledge of the intervention received by study participants |
| Selective reporting (reporting bias) | Low risk | All reported results correspond to all intended outcomes |
| Haddad et al. 2022 | Risk of bias | Author judgement |
| Confounding | Low risk | No confounding expected |
| Allocation concealment (selection bias) | Low risk | All participants who would have been eligible for the target trial were included in the study |
| Incomplete outcome data (attrition bias) | Low risk | Data was complete |
| Blinding of outcome assessment (detection bias) | Low risk | The methods of outcome assessment were comparable across intervention groups; The outcome measure is only minimally influenced by knowledge of the intervention received by study participants |
| Selective reporting (reporting bias) | Low risk | All reported results correspond to all intended outcomes |
| Huang et al. 2019 | Risk of bias | Author judgement |
| Confounding | Low risk | No confounding expected |
| Allocation concealment (selection bias) | Low risk | All participants who would have been eligible for the target trial were included in the study. For each participant, start of follow up and start of intervention coincided |
| Incomplete outcome data (attrition bias) | Low risk | Data was complete |
| Blinding of outcome assessment (detection bias) | Low risk | The methods of outcome assessment were comparable across intervention groups; The outcome measure is only minimally influenced by knowledge of the intervention received by study participants |
| Selective reporting (reporting bias) | Low risk | All reported results correspond to all intended outcomes |
| Koemans et al. 2021 | Risk of bias | Author judgement |
| Confounding | Low risk | No confounding expected |
| Allocation concealment (selection bias) | High risk | TRG3 patients were excluded, which was related to intervention and outcome |
| Incomplete outcome data (attrition bias) | Low risk | Data was complete |
| Blinding of outcome assessment (detection bias) | High risk | TRG3 patients were initially excluded. However, following the initial analysis, TRG3 patients got included for some comparisons. Therefore, the outcome measure was subjective and the outcome was assessed by assessors aware of the intervention received by study participants |
| Selective reporting (reporting bias) | Low risk | All reported results correspond to all intended outcomes |
| Kotsafti et al. 2020 | Risk of bias | Author judgement |
| Confounding | Low risk | No confounding expected |
| Allocation concealment (selection bias) | Low risk | All participants who would have been eligible for the target trial were included in the study. For each participant, start of follow up and start of intervention coincided |
| Incomplete outcome data (attrition bias) | Low risk | Data was complete |
| Blinding of outcome assessment (detection bias) | Low risk | The methods of outcome assessment were comparable across intervention groups; The outcome measure is only minimally influenced by knowledge of the intervention received by study participants |
| Selective reporting (reporting bias) | High risk | The selected genes described as possible markers for immune microenvironment in the method section are not all described in the results |
| Kukar et al. 2020 | Risk of bias | Author judgement |
| Confounding | High risk | The intervals between the first and second PETCT differed significantly between patients; The intervals between last radiation therapy and second PETCT differed between patients. These time differences were not controlled for in the analyses |
| Allocation concealment (selection bias) | Low risk | All participants who would have been eligible for the target trial were included in the study. For each participant, start of follow up and start of intervention coincided |
| Incomplete outcome data (attrition bias) | Low risk | Data was complete |
| Blinding of outcome assessment (detection bias) | Low risk | All assessors were blinded |
| Selective reporting (reporting bias) | Low risk | All reported results correspond to all intended outcomes |
| Lee et al. 2021 | Risk of bias | Author judgement |
| Confounding | Low risk | No confounding expected |
| Allocation concealment (selection bias) | Low risk | All participants who would have been eligible for the target trial were included in the study; For each participant, start of follow up and start of intervention coincided |
| Incomplete outcome data (attrition bias) | Low risk | Data was complete |
| Blinding of outcome assessment (detection bias) | Low risk | The methods of outcome assessment were comparable across intervention groups; The outcome measure is only minimally influenced by knowledge of the intervention received by study participants |
| Selective reporting (reporting bias) | Low risk | There is no indication of selection of the reported analysis from among multiple analyses; The outcome measurements are clearly defined and both internally and externally consistent |
| Li et al. 2021 | Risk of bias | Author judgement |
| Confounding | Low risk | No confounding expected |
| Allocation concealment (selection bias) | Low risk | All participants who would have been eligible for the target trial were included in the study. For each participant, start of follow up and start of intervention coincided |
| Incomplete outcome data (attrition bias) | Low risk | Data was complete |
| Blinding of outcome assessment (detection bias) | Low risk | The methods of outcome assessment were comparable across intervention groups; The outcome measure is only minimally influenced by knowledge of the intervention received by study participants |
| Selective reporting (reporting bias) | Low risk | There is no indication of selection of the reported analysis from among multiple analyses; The outcome measurements are clearly defined and both internally and externally consistent |
| Piessen et al. 2013 | Risk of bias | Author judgement |
| Confounding | Low risk | No confounding expected |
| Allocation concealment (selection bias) | Low risk | All participants who would have been eligible for the target trial were included in the study; For each participant, start of follow up and start of intervention coincided |
| Incomplete outcome data (attrition bias) | Low risk | The analysis addressed missing data and is likely to compensate for any risk of bias |
| Blinding of outcome assessment (detection bias) | Low risk | Methods of outcome assessment were comparable between both groups; the outcome measure was unlikely to be influenced by knowledge of the intervention received by study participants (blinded) |
| Selective reporting (reporting bias) | Low risk | All reported results correspond to all intended outcomes |
| Soeratram et al. 2021 | Risk of bias | Author judgement |
| Confounding | Low risk | No confounding expected |
| Allocation concealment (selection bias) | Low risk | All participants who would have been eligible for the target trial were included in the study; For each participant, start of follow up and start of intervention coincided |
| Incomplete outcome data (attrition bias) | Low risk | Data was complete |
| Blinding of outcome assessment (detection bias) | Low risk | Methods of outcome assessment were comparable between both groups; the outcome measure was unlikely to be influenced by knowledge of the intervention received by study participants |
| Selective reporting (reporting bias) | Low risk | All reported results correspond to all intended outcomes |
| Van Rossum et al. 2017 | Risk of bias | Author judgement |
| Confounding | High risk | Patients received various kinds of chemoradiotherapy which were not controlled for in the analyses |
| Allocation concealment (selection bias) | Low risk | All participants who would have been eligible for the target trial were included in the study; For each participant, start of follow up and start of intervention coincided |
| Incomplete outcome data (attrition bias) | Low risk | The analysis addressed missing data and is likely to have removed any risk of bias |
| Blinding of outcome assessment (detection bias) | Low risk | Methods of outcome assessment were comparable between both groups; the outcome measure was unlikely to be influenced by knowledge of the intervention received by study participants |
| Selective reporting (reporting bias) | High risk | There is a high risk of selective reporting from among multiple analyses as three patients were excluded from the survival analyses without further explanation |
| Wang et al. 2010 | Risk of bias | Author judgement |
| Confounding | Low risk | No confounding expected |
| Allocation concealment (selection bias) | Low risk | All participants who would have been eligible for the target trial were included in the study; For each participant, start of follow up and start of intervention coincided |
| Incomplete outcome data (attrition bias) | Low risk | The analysis addressed missing data and is likely to have removed any risk of bias |
| Blinding of outcome assessment (detection bias) | Low risk | Methods of outcome assessment were comparable between both groups; the outcome measure was unlikely to be influenced by knowledge of the intervention received by study participants |
| Selective reporting (reporting bias) | Low risk | All reported results correspond to all intended outcomes |
